# Correlation between anti-malarial and anti-haemozoin activities of anti-malarial compounds

**DOI:** 10.1186/s12936-020-03370-x

**Published:** 2020-08-21

**Authors:** Dao Ngoc Hien Tam, Gehad Mohamed Tawfik, Amr Ehab El-Qushayri, Ghaleb Muhammad Mehyar, Sedralmontaha Istanbuly, Sedighe Karimzadeh, Vo Linh Tu, Ranjit Tiwari, Truong Van Dat, Phuong Thuy Viet Nguyen, Kenji Hirayama, Nguyen Tien Huy

**Affiliations:** 1 Asia Shine Trading & Service CO. LTD., Ho Chi Minh City, Vietnam; 2 Online Research Club, Nagasaki, Japan; 3 Faculty of Medicine, Ain Shams University, Cairo, Egypt; 4 Faculty of Medicine, Minia University, Minia, 61519 Egypt; 5 Southwest Physicians Associates S.C., 2955 W 95th St, Evergreen Park, IL 60805 USA; 6 Faculty of Medicine, University of Aleppo, Aleppo, Syrian Arab Republic; 7 School of Medicine, Sabzevar University of Medical Sciences, Sabzevar, Iran; 8 University of Medicine and Pharmacy at Ho Chi Minh City, Ho Chi Minh City, Vietnam; 9 Faculty of Medicine, Institute of Medicine, Tribhuvan University, Kathmandu, 44600 Nepal; 10 Department of Immunogenetics, Institute of Tropical Medicine (NEKKEN), Leading Graduate School Program, and Graduate School of Biomedical Sciences, Nagasaki University, 1-12-4 Sakamoto, Nagasaki, 852-8523 Japan; 11 School of Tropical Medicine and Global Health, Nagasaki University, 1-12-4 Sakamoto, Nagasaki, 852-8523 Japan; 12 Institute of Research and Development, Duy Tan University, Da Nang, 550000 Vietnam

**Keywords:** Malaria, Anti-haemozoin, Correlation, Systematic review

## Abstract

**Background:**

Despite noticeable improvement in anti-malarial treatment, rapid growth of resistant malaria strains points out the need for continuous development of novel anti-malarials to fight the disastrous infection. Haemozoin is considered as a novel inhibitory pathway for new anti-malarial drugs, therefore, this study aimed to systematically review all articles investigating the correlation between anti-malarial and anti-haemozoin activities of anti-malarial compounds.

**Methods:**

A literature search was conducted on 22 October 2017 in eight databases for relevant in vitro articles reporting the correlation between anti-malarial and anti-haemozoin of anti-malarial compounds, based on the constructed search terms and inclusion criteria. ToxRtool was used to assess quality of each study.

**Results:**

A total of ten articles were included in the review. In vitro anti-malarial and anti-haemozoin activity had a good correlation for quinolines for sensitive strains (R^2^ ranging from 0.66 to 0.95) and xanthones (Spearman ρ = 0.886). However, these correlations were reached after removing some compounds which had non-detectable anti-malarial or anti-haemozoin effects. Other structures (acridines, pyrolidines) showed negligible correlation with Spearman ρ ranging from 0.095 to 0.381 for acridines, and r varying from 0.54 to 0.62 for pyrolidines. Some good correlations were only shown in a logarithmic manner or when the anti-malarial activity was normalized.

**Conclusion:**

The results raised a relative relationship between anti-haemozoin and in vitro anti-malarial activities. Some studies reported compounds that were effective in the inhibition of haemozoin formation, but failed to inhibit the parasite survival and vice versa. The correlation results in these studies were calculated after these compounds were removed from their analysis. The ability of anti-malarial compounds to accumulate inside the reaction site might strengthen their anti-malarial activity.

## Background

Malaria incidence has been recently decreasing, however, it still remains one of the most dangerous parasitic infections that accounted for 405,000 deaths in 2018. Five parasites of the genus *Plasmodium,* namely *Plasmodium falciparum, Plasmodium vivax, Plasmodium malariae, Plasmodium knowlesi,* and *Plasmodium ovale*, are responsible for malaria in humans [1, 2]. Malaria includes a wide spectrum of clinical manifestations, such as fever, headache and chills after host blood invasion; however, severe malaria may progress to death mainly from severe anemia if not medically controlled [3, 4]. Chloroquine (CQ) has been an anti-malarial agent since 1940s as an efficient treatment with a wide safety margin and lower reported side effects [5]. Recently, it has been replaced by artemisinin-based combination therapy (ACT) as a widely used anti-malarial treatment. However, signs of resistance to artemisinin-based combinations have been reported leading to the need of new anti-malarial agents fighting malaria infection [6, 7]. Despite the progress in treatment, management of malaria remains a critical issue among physicians due to the lack of commercial malaria vaccine and the emergence of drug-resistant strains hindering anti-malarial drugs action. That can be resulted from the misuse of anti-malarial drugs for the benefit of prophylaxis, monotherapy usage, and the reduced application of effective therapy for malarial patients [1, 8–14].

The digestion of haemoglobin inside the parasites results in an end product called haem, which is lethal to them. Due to the absence of haem oxygenase enzyme, the parasite depends on the haem detoxification system which includes the haem dimerization inside food vacuole to form haemozoin pigment, and other processes involved in reduced glutathione, peroxidase or haem binding proteins [15–18]. Haemozoin is identical to β-haematin—a crystallized haem dimer which could be created by chemical synthesis using haemin [19, 20]. The haemozoin could be formed by malarial preformed haemozoin, proteins or lipids, however, its mechanism has not yet clarified [18]. Nevertheless, the interruption of the haem crystallization in parasitic food vacuole is still considered an effective way to enhance free haem toxicity, and the screening of these inhibitors could be a novel orientation for anti-malarial drugs discovery [15–18].

The correlation between in vitro anti-malarial the anti-haemozoin activities of quinolines, acridines has been reported in various studies [21–24]. These reports demonstrated significant correlations between in vitro anti-plasmodial activity of quinolines as well as their role in inhibition of β-haematin synthesis while acridines had no correlation between them. The strong correlation implies the potent of β-haematin inhibitors as anti-malarial candidates with their probable mechanism of anti-malarial action via the inhibition of crystallization process. Several studies also showed that various anti-malarial agents with high efficacy, such as azoles, xanthones also possessed the β-haematin inhibition [1, 18, 21, 25, 26]. To have a thorough perspective relating to the probable efficacy of anti-haemozoin compounds so that more anti-malarial candidates could be screened, this study aimed to investigate the correlation between anti-malarial and anti-haemozoin activity by conducting a systematic review. This study may provide useful information about the efficacy of targeting haemozoin formation pathway in anti-malarial drug discovery.

## Methods

### Search strategy and study selection

The study was conducted following the accepted methodology recommendations of PRISMA’s (Preferred Reporting Items for Systematic reviews and Meta-Analyses) checklist for systematic reviews (Additional file 1: Table S1) [27]. The steps were done following a guideline, which was published elsewhere [28]. The protocol of this study was presented in ResearchGate (10.13140/rg.2.2.16654.41289).

A search in systematic electronic databases for suitable studies on 22 October 2017 in eight databases including Google Scholar, Popline, WHO health library (GHL), System for Information on Grey Literature in Europe (SIGLE), Scopus, Web of science (ISI), PubMed, Virtual Health Library (VHL) using the following search terms: (hemozoin OR haemozoin OR hematin OR haematin OR (heme polymer) OR (haem polymer) OR (heme polymerization) OR (haem polymerization)) AND (antimalarial OR antimalaria OR antiplasmodial OR antimalarial OR (anti-plasmodial) OR (anti plasmodium) OR antiplasmodial) AND (correlation OR correlated OR association OR relation). Furthermore, a manual search was conducted by searching references from included articles by searching the primary studies that had cited included articles and scanning the relevant papers in Google Scholar and PubMed to avoid missing any relevant publication. The inclusion criteria were (i) studies investigating correlation between anti-haemozoin and anti-malarial drugs (in vitro studies) and (ii) no restriction on study design, country, languages or publication date. Exclusion criteria were studies reporting unreliable data for extraction.

**Fig. 1:**
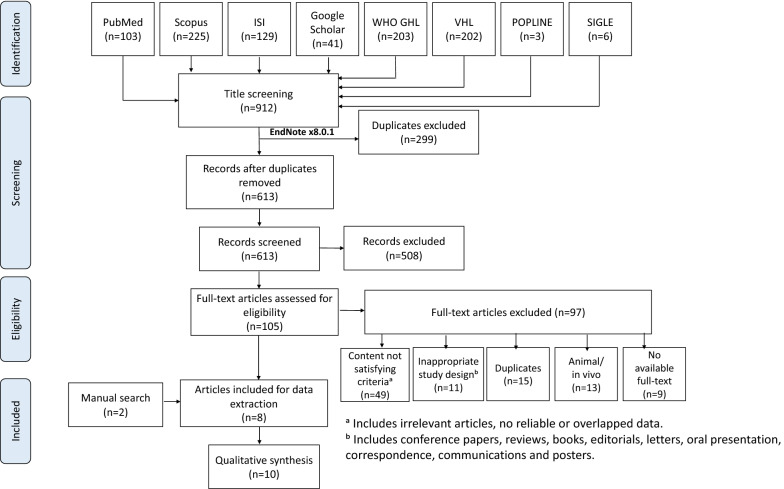
PRISMA flow diagram showing the process of the review

Three reviewers independently screened title and abstract of searched articles for their initial inclusion. Full-texts of eligible articles were then collected for full-text screening step. All original articles met our criteria were included for qualitative analysis. In both screening steps, inclusion or exclusion of a study was agreed by three independent reviewers. A discussion between them was made to reach the final conclusion if there were any disagreements. When necessary, senior reviewers were consulted to address any discrepancies. The study selection procedure is summarized in the PRISMA flow diagram (Fig. 1).

### Data extraction

Based on a pilot review of a random five papers, a data extraction form was developed by two authors. The form was divided into three parts. The first part included study characteristic data. The second part included correlation results of anti-malarial and anti-haemozoin (Pearson’s r, Spearman’s ρ or R^2^), the half maximal inhibitory concentration (IC_50_) values of anti-malarial activity, the normalized-IC_50_ values of anti-malarial activity which were estimated by multiplying IC_50_ values and the relative vacuolar accumulation ratio, and IC_50_ values of anti-haemozoin activity (β-haematin inhibitory activity 50%, BHIA_50_). The third part was used to assess quality of each study. Data were extracted by three reviewers independently by a pre-structure excel sheet. All disagreements and discrepancies were resolved by discussion and consensus.

For papers that only reported the conclusion of the correlation but there were no correlation results reported, their recalculation was performed by SPSS (version 25.0, Armonk, NY, IBM Corp.).

The main outcome was the correlation result of scaffolds between anti-malarial and anti-haemozoin activities analyzed by correlation tests such as Pearson, Spearman or R^2^. No analysis to pool all correlation results was conducted due to the heterogeneity across the studies.

### Quality assessment

Three independent reviewers evaluated risk of bias in included studies. Methodological quality assessment was done using ToxRTool quality assessment tool [29]. The tool consists of 18 questions (for in vitro studies) to evaluate quality of each study. There are critical questions that are suggested as the most important elements relating to the quality of a study, with each “yes” answer for each question, the study gets 1 point. Studies obtained score of (15–18) were considered reliable without restrictions, (11–14) reliable with restrictions and (< 11) were considered not reliable. However, if there were any critical questions with “no” answer, the level of quality could be downgraded. The excel file containing explanation and points for each category could be downloaded via this website: https://ec.europa.eu/jrc/en/scientific-tool/toxrtool-toxicological-data-reliability-assessment-tool?fbclid=IwAR3mCgiQlVzIkhBVP0XQV7Gq6uNjhwccE2VROdBD7rnj5WLa6hbkDRgvMGg. The overall quality of each study was concluded by discussion and consensus between three reviewers.

## Results

### Study selection

The initial search included 912 articles after searching on selected databases. Of 912 articles, 613 records were yielded for screening title and abstract after removing 299 duplicates via Endnote software. Then, there were 105 articles that were eligible for full text screening and eight studies of which were subsequently included. Additional two articles were added as a result of manual search trials. Finally, ten in vitro studies were included for this systematic review. Details of each step and the reasons of exclusion were presented in Fig. 1.

### Characteristics of selected studies

Of ten included studies, five studies investigated the anti-malarial and anti-haemozoin activities of quinolines [22, 23, 30–32] (Table 1). Others examined these activities of biquinolines, pyrazolines, xanthones, and acridines separately. Only one study reported the correlation between the anti-malarial activity of varied chemical groups (phenyl quinolines, phenyl benzamides, triaryl imidazole, benzylethene) and their anti-haemozoin activity, and one study showed the correlation between in vitro anti-malarial activity of one group of compounds on different strains and the anti-haemozoin activity [30, 33]. The *Plasmodium falciparum* strains studied were both sensitive and resistant strains. The majority of experiments used high concentration of acetate [21, 24, 32] and preformed β-haemozoin assay [23, 31, 34]. Few studies used the methods of heat-induced β-haematin, tween 20-induced β-haematin, phosphate-induced β-haematin and NP-40-induced β-haematin [22, 30, 33, 35].

**Table 1:** Characteristic of included studies

Author/Year/Country	Chemical groups	*Plasmodium falciparum* strain	Anti-hemozoin assay	Correlation test	Correlation results	Risk of bias
Acharya/2010/India [21]	Pyrazoline	Chloroquine-sensitive MRC-02	High concentration of acetate	Pearson’s r	r = 0.62^a^	Reliable without restrictions
	Pyrazoline (+chloroquine)				r = 0.63^a^	
	Pyrazoline	Chloroquine resistant RKL9			r = 0.54^a^	
	Pyrazoline (+chloroquine)				r = 0.40^a^	
Andayi/2013/South Africa [22]	Quinoline (chloroquine deprotected analogues)	Chloroquine sensitive 3D7	Heat-induced β-haematin	R^2^	R^2^ = 0.92	Reliable with restrictions
				Spearman	ρ = 0.595 (p = 0.12)	
		Chloroquine resistant W2		R^2^	R^2^ = 0.95	
				Spearman	ρ = 0.976 (p = 0.00003)	
		Chloroquine resistant K1		Spearman	ρ = 0.643 (p = 0.119)	
	Quinoline (chloroquine benzylated analogues)	Chloroquine sensitive 3D7		R^2^	R^2^ = 0.96	
				Spearman	ρ = 0.464 (p = 0.294)	
		Chloroquine resistant W2		R^2^	R^2^ = 0.93	
				Spearman	ρ = 0.821 (p = 0.023)	
		Chloroquine resistant K1		Spearman	ρ = 0.571 (p = 0.139)	
	Quinoline (chloroquine methoxy analogues)	Chloroquine resistant W2		Spearman	ρ = -0.600 (p = 0.6)	
Hawley/1998/UK [23]	Quinoline	Chloroquine sensitive 3D7	Preformed β-haemozoin	Pearson’s r	r = 0.81	Reliable with restrictions
		Chloroquine resistant K1			r = 0.17	
Burgess/2010/USA [30]	Quinoline (Reversed chloroquinolines)	Chloroquine sensitive D6	Tween 20-induced β-haematin	R^2^	R^2^ = 0.66	Not reliable
				Spearman	ρ = 0.476 (p = 0.233)	
		Chloroquine resistant Dd2		Spearman	ρ = 0.574 (p = 0.183)	
		Chloroquine resistant 7G8		Spearman	ρ = 0.333 (p = 0.42)	
Vennerstrom/1998/USA [34]	Biquinolines	Chloroquine sensitive D6	Preformed β-haemozoin	Pearson’s r	r = 0.29	Not reliable
		Chloroquine resistant W2			r = 0.55	
		Chloroquine sensitive D6 Chloroquine resistant W2			r = 0.57	
		Chloroquine sensitive D6		Spearman	ρ = 0.376 (p = 0.254)	
		Chloroquine resistant W2			ρ = 0.67 (p = 0.024)	
		Chloroquine sensitive D6 Chloroquine resistant W2			ρ = 0.519 (p = 0.013)	
Dorn/1998/Switzerland [31]	Quinolines, quinacrine and halofantrine	Quinoline sensitive NF54	Preformed β-haemozoin	Pearson’s r	r = 0.919 (p = 0.003)	Reliable with restrictions
Kaschula/2002/South Africa [32]	Quinoline	Chloroquine sensitive D10	High concentration of acetate -	R^2^	R^2^ = 0.83^a^	Reliable with restrictions
		Chloroquine sensitive D10		Spearman	ρ = 0.919^a^	
Ignatushchenko/1997/USA [35]	Xanthone	Chloroquine sensitive D6	Phosphate-induced β-haematin	Spearman	ρ = 0.886 (p = 0.019)	Reliable with restrictions
Guetzoyan/2009/France [24]	Acridine	Chloroquine sensitive 3D7	High concentration of acetate	Spearman	ρ = 0.095 (p = 0.823)	Reliable with restrictions
		Chloroquine sensitive W2			ρ = 0.299 (p = 0.471)	
		Chloroquine resistant FCR3			ρ = 0.381 (p = 0.352)	
		Chloroquine resistant Bre1			ρ = 0.261 (p = 0.531)	
Sandlin/2014/USA [33]	Quinoline 4-benzamidopyridine Quinazoline Phenyl benzamides Nicotinamide Carbazole Miscellaneous compounds	Chloroquine sensitive D6	NP-40-induced β-haematin	Spearman	ρ = 0.35 (p = 0.322)	Reliable with restrictions
		Chloroquine sensitive C235			ρ = 0.139 (p = 701)	

^a^The logarithmic correlation

### Risk of bias of included studies

In terms of risk of bias, one study was qualified as reliable without restrictions, seven studies as reliable with restrictions and two studies as not reliable (Table 1).

### Correlation between anti-haemozoin and anti-malarial activity

**Fig. 2:**
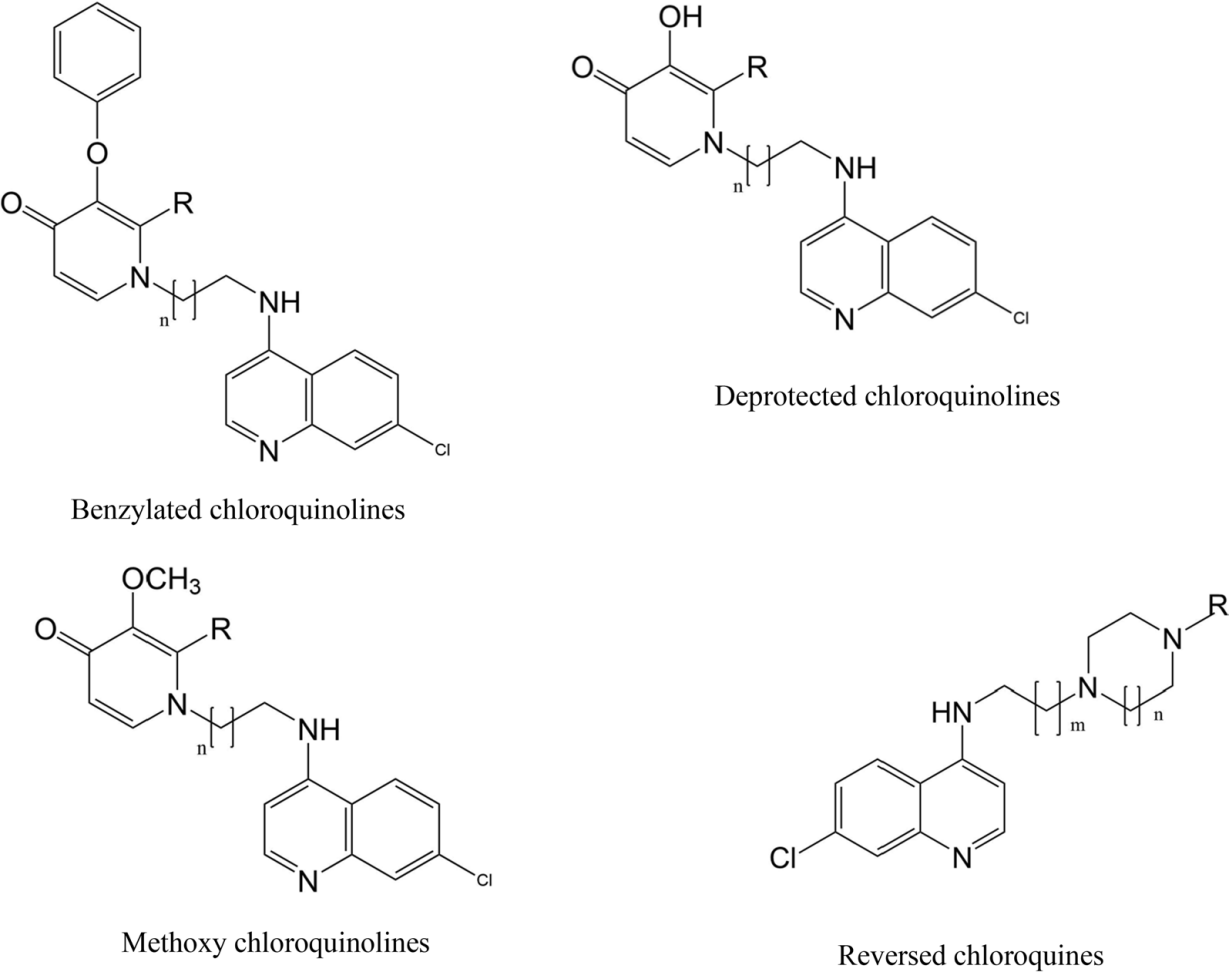
Structure of benzylated chloroquinolines, deprotected chloroquinolines, methoxy chloroquinolines and reversed chloroquines

There were six studies that investigated the correlation between in vitro anti-malarial activity of quinoline compounds and anti-haemozoin activity [22, 23, 30–32, 34]. Three of them studied chloroquinoline analogues. The correlations between the activities of chloroquinolines were varied according to *P. falciparum* strains. Against sensitive strain 3D7 and sensitive strain D6, the quite good correlations between normalized-IC_50_ values of in vitro anti-malarial activity and IC_50_ values of anti-haemozoin activity were respectively reported by Pearson’s r = 0.81 and R^2^ = 0.66 (Spearman ρ = 0.476, p = 0.233, Additional file 2: Fig. S1 and Additional file 3: Fig. S2) [23, 30]. The normalized-IC_50_ value was calculated by multiplying the absolute IC_50_ value with the vacuolar accumulation ratio of the compound to compensate the differences of activities between compounds caused by their varied accumulated concentration in the food vacuoles. For the correlation between these activities of reversed chloroquines (Additional file 2: Fig. S1 and Additional file 3: Fig. S2), a compound that possessed a strong in vitro anti-malarial activity (IC_50_ = 2–10 nM) was removed from the analysis due to its insoluble form in anti-haemozoin test [30]. Both benzylate chloroquinolines and deprotected chloroquinolines (without benzylate moiety) (Fig. 2) also showed strong correlations between IC_50_ values of in vitro anti-malarial activity in sensitive strain 3D7 and IC_50_ values of anti-haemozoin activity with R^2^ = 0.92 (Spearman ρ = 0.595, p = 0.12) and 0.96 (Spearman ρ = 0.464, p = 0.294), respectively [22] (Additional file 4: Fig. S3, Additional file 5: Fig. S4, Additional file 6: Fig. S5 and Additional file 7: Fig. S6). A dihydrochloride salt of a benzylate chloroquine (compound 8d) was removed from this analysis due to its inactivation of haemozoin formation despite having good anti-malarial effect. The in vitro anti-malarial activity of these analogues also significantly correlated with the inhibition of haemozoin formation in resistant strain W2, by R^2^ = 0.95 (Spearman ρ = 0.976, p = 0.00003) and 0.93 (Spearman ρ = 0.821, p = 0.023) (Additional file 8: Fig. S7, Additional file 9: Fig. S8, Additional file 10: Fig. S9 and Additional file 11: Fig. S10). However, the correlations were poorer for resistant strains (K1, 7G8). For K1 strain, Pearson’s r was 0.17 for chloroquinolines (amodiaquine, chloroquine, monodesethyl amodiaquine, *N*-t-butyl amodiaquine, amopyroquine, dehydroxytebuquine, 4′-dehydroxy-4′-fluorotebuquine); and Spearman ρ ranged from 0.571 (p = 0.139) to 0.643 (p = 0.119) for deprotected chloroquinoline analogues and benzylated analogues (Additional file 12: Fig. S11, Additional file 13: Fig. S12, Additional file 14: Fig. S13 and Additional file 15: Fig. S14) [22, 23]. A benzylate chloroquine (compound 8d) was also removed from this analysis because of its non-detectable anti-haemozoin activity. Similarly, reversed chloroquines (Fig. 2) in Burgess’s report showed the Spearman ρ = 0.333 (p = 0.42) for 7G8 strain (Additional file 16: Fig. S15 and Additional file 17: Fig. S16). The exceptional case was for resistant Dd2 strain with Spearman ρ = 0.574 (p = 0.183) (Additional file 18: Fig. S17 and Additional file 19: Fig. S18) [30].

Similar to chloroquinolines, other quinolines had in vitro anti-malarial activity strongly correlated with the anti-haemozoin activity [31, 32] as demonstrated by the high correlation coefficient (Pearson’s r = 0.919) between the IC_50_ values against strain NF54 and BHIA_50_ values [31]. However, it must be noted that there were two compounds of other scaffolds besides five quinolines in this analysis, namely quinacrine and halofantrine. For strain D10, Kaschula et al. [32] found that a strong correlation was only seen between log of normalized-IC_50_ of the inhibition of parasite growth and log of BHIA_50_ [R^2^ = 0.83, Spearman ρ = 0.919 (p = 0.003)] (Additional file 20: Fig. S19), while there was no correlation found between log of IC_50_ values and log of BHIA_50_ (R^2^ = 0.08). There were three compounds (with hydro-, hydroxyl-, and acetyl-radicals) removed from this analysis, because they had very weak in vitro anti-malarial activity (IC_50_ ranging from 448 to 3017 nM) and non-detectable anti-haemozoin activity. In contrast, the correlations between these activities (IC_50_ values of anti-malarial and anti-haemozoin activities) were only modest for biquinoline analogues [34]. Precisely, the highest correlation reported in this study was Pearson’s r = 0.61 if only the lowest IC_50_ values against two strains (W2 and D6) were collected. The correlation coefficient (Pearson’s r) slightly decreased to 0.55 (Spearman ρ = 0.67, p = 0.024, Additional file 21: Fig. S20 and Additional file 22: Fig. S21) in W2 strain individually, or was 0.57 (Spearman ρ = 0.519, p = 0.013, Additional file 23: Fig. S22 and Additional file 24: Fig. S23) when the authors pooled the data of average IC_50_ against both D6 and W2 strains. This correlation was even very poor [r = 0.29, (Spearman ρ = 0.376, p = 0.254), Additional file 25: Fig. S24 and Additional file 26: Fig. S25] between IC_50_ of in vitro anti-malarial activity of strain D6 alone and IC_50_ of the inhibition of haematin formation.

Pyrazolines, xanthones and acridines were individually studied with varied results of correlation [21, 24, 35]. The correlation between IC_50_ values of in vitro anti-malarial activity of xanthones in D6 and IC_50_ values of their haem formation inhibition was highest amongst these studies (Spearman ρ = 0.886, p = 0.019), as shown in Additional file 27: Fig. S26 and Additional file 28: Fig. S27. 2-Hydroxanthone, 1,3-dihydroxyxanthone, and 2,3,4,5,6- pentaacetylxanthone were active against *P. falciparum* D6 strain (IC_50_ ranged from 0.075 to 75 µM) but did not possess the anti-haemozoin activity (IC_50_ > 1000 µM). Therefore, they were not included in the analysis. The in vitro anti-malarial activity (log of normalized-IC_50_) of pyrazolines had a moderate correlation with the anti-haemozoin activity (log of BHIA_50_) with Pearson’s r = 0.62 (for sensitive MRC-02 strain) and Pearson’s r = 0.63 (for resistant RKL9 strain) [21]. These correlation coefficient values decreased to 0.54 and 0.40, respectively, when adding the results of chloroquine phosphate, possibly because of the resistance of RKL9 strain against chloroquine. Finally, the acridines activity (IC_50_) against four strains (3D7, W2, FCR3, Bre1) separately had no tight correlation with the anti-haemozoin activity (BHIA_50_), as the Spearman ρ ranged from 0.095 to 0.381 (p > 0.05) [24]. These correlations were shown in Additional file 29: Fig. S28, Additional file 30: Fig. S29, Additional file 31: Fig. S30, Additional file 32: Fig. S31. The low correlations [Spearman ρ = 0.35 (p = 0.322), and 0.139 (p = 0.701)] between BHIA_50_ and IC_50_ of in vitro anti-malarial activity (in chloroquine-resistant D6 strain and multidrug-resistant strain, respectively) were also revealed in Additional file 33: Fig. S32 and Additional file 34: Fig. S33, when studying the activity of scaffolds of quinoline, 4-benzamidopyridine, quinazoline, phenyl benzamides, nicotinamide, carbazole and 2 miscellaneous compounds which could not be classified in a specific scaffold [33].

Exceptionally, there was a case showing negative correlation between in vitro anti-malarial activity and anti-haemozoin activity. The Spearman ρ was − 0.6 (p = 0.6) for chloroquinoline methoxy analogues (Fig. 2) against resistant strain W2. Two methoxy chloroquines also showed no affection on sensitive strain 3D7 and resistant strain K1, although their BIHA_50_ values ranged from 0.47 to 1.71. On the other hand, artemisinin analogues (arteether, dihydroartemisinine, artemether) were strong anti-malarial compounds, but had no effect on haemozoin formations, indicating another mechanism of action differing from anti-haemozoin activity [23].

A summarized of correlation results and characteristics of groups compound were shown in Table 2.

**Table 2:** Highlight features of chemical groups relate to the correlation between anti-hemozoin and in vitro antimalarial activity

Chemical groups	Characteristics
Quinoline	Strong correlations between anti-haemozoin and in vitro anti-malarial activity, against sensitive strain (NF54), r = 0.919 (p = 0.003). Logarithm manner of the correlation observed between BHIA_50_ and normalized-IC_50_ values of in vitro anti-malarial activity in strain 3D10 (R^2^ = 0.83, Spearman ρ = 0.919) There were three compounds (with hydro-, hydroxyl-, and acetyl-radicals at 7-position) were removed from the analysis, because they had very weak anti-malarial activity (IC_50_ ranging from 448 to 3017 nM) and non-detectable anti-haemozoin activity Strong affinity with haem
Quinoline (chloroquinoline)	Quite good or strong correlation between anti-haemozoin and in vitro anti-malarial activity, against sensitive strains 3D7 (R^2^ = 0.92–0.96, Spearman ρ = 0.464–0.595), resistant strain W2 (R^2^ = 0.93–0.95, Spearman ρ = 0.821–0.976). The good correlations between these two activities on 3D7 strain might be fortuitous Poorer correlations recorded for resistant strain K1 (Spearman ρ = 0. 571–0.643) A compound was removed from this analysis due to its inactivation of haemozoin formation, despite it might have good anti-malarial effect
Quinoline (Reversed chloroquinoline)	Pretty good correlation observed when normalizing the IC_50_ values of in vitro anti-malarial activity, against sensitive strain D6 (R^2^ = 0.66, Spearman ρ = 0.476 (p = 0.233)), and resistant strain Dd2 (Spearman ρ = 0.574 (p = 0.183)) Poorer correlation observed on resistant strain 7G8 (Spearman ρ = 0.333 (p = 0.42)) A compound which had strong anti-malarial activity (IC_50_ = 2 nM) was removed from the analysis due to its insoluble form in anti-haemozoin test
Xanthone	Good correlation between anti-haemozoin and in vitro anti-malarial activity in strain D6 (Spearman ρ = 0.886 (p = 0.019)) Strong affinity with haem Position 4-, and 5- in xanthone scaffold were favored to the good correlation Three compounds were removed from the analysis, namely 2-hydroxanthone, 1,3-dihydroxyxanthone, 2,3,4,5,6- pentaacetylxanthone. They were active against *P. falciparum* D6 strain (IC_50_ ranged from 0.075 to 75 µM) but did not possess the anti-haemozoin activity (IC_50_ > 1000 µM)
Quinoline (biquinoline)	Modest correlation with pooled data for strains W2, D6 (R = 0.57, Spearman ρ = 0.519 (p = 0.013)) Poor correlation for strain D6 (R = 0.29, Spearman ρ = 0.376 (p = 0.254))
Pyrazoline	Modest correlation (r = 0.54–0.62) Logarithm manner of the correlation observed between normalized-IC_50_ values of in vitro anti-malarial activity (MRC-02 strain and RKL9 strain) and BHIA_50_. Lower correlation values happened when adding chloroquine
Acridine	No correlation observed on strains 3D7, W2, FCR3, Bre1 (Spearman ρ = 0.095, 0.299, 0.381, 0.261, respectively)

## Discussion

From the literature, the study found that chloroquinoline derivatives, quinolines and xanthone derivatives respectively had strong correlations between the in vitro anti-malarial activity and anti-haemozoin activity, in sensitive strains (3D7, NF54, D6, D10) and resistant strain (W2). The high correlation for chloroquinolines were consistent in 3D7 strain [23, 30]. Biquinolines and pyrazolines’ in vitro anti-malarial activity only had modest correlation with their anti-haemozoin activity [21, 34]. Finally, acridines did not show any correlation between their in vitro anti-malarial and anti-haemozoin activities, against four individually resistant strains [24].

In the food vacuoles, parasites digest the haemoglobin for several reasons [18, 36]. As a result of the haemoglobin digestion, free haem is released and could lead to the parasite death via the oxidative stress or the changes in membrane permeability. That why the parasites need to detoxify free haem via the haemozoin formation for their survival. The degradation occurs inside the food vacuole of the parasite, where is acidic with pH of 5.0–5.4 [36]. This condition inside the parasite was maintained via a proton gradient regulated by an ATPase pump. Chloroquine, an existing malarial drug, was also known as a β-haematin inhibitor. These molecules entered the food vacuole by simple diffusion, then remained inside this acidic environment by the protonation, as this compound had the high pKa value. It should be emphasized that the diprotonic form prevented chloroquine from diffusing back. The results reported by Kaschula et al. [32] showed the evidence relating to the role of pH trapping to the correlation between in vitro anti-malarial and anti-haemozoin activities, as the correlation between these activities was only observed when IC_50_ values were normalized by multiplying with the vacuolar accumulation. Otherwise, absolute IC_50_ values and BHIA_50_ were very week correlated.

In our study, we observed the relatively good correlations between normalized-IC_50_ values of in vitro anti-malarial activity and IC_50_ values of anti-haemozoin activity when examining several compounds of chloroquinoline analogues (amodiaquine, chloroquine, amodesethyl amodiaquine, *N*-tert-butyl amodiaquine, amopyroquine, 4′-dehydroxytebuquine), some reversed chloroquinolines, and pyrazolines [21, 23, 30]. This correlation was even logarithmic for some quinolines. Also, the poor correlations between IC_50_ value and BIAH_50_ was even revealed, for example, R^2^ = 0.08 for the correlation [23, 32]. These results revealed that not all of these compounds accumulated enough in the food vacuoles to thoroughly cause the inhibition of haemozoin formation as shown in vitro, and their ability of the accumulation in the food vacuoles were varied as well. This variation related to their varied pKa values, which made their concentration differently distributed in the food vacuoles inside the parasites, and then the normalization compensated the differences. These articles also recorded the wide ranges of vacuoles accumulation ratio and the amount of their un-ionic forms inside the food vacuoles.

This theory could also be applied to explain the poor correlation between anti-haemozoin activity and in vitro anti-malarial activity of acridine scaffold and the modest correlation between these two activities of biquinolines [24, 34]. Guetzoyan et al. [24] reported that acridines with higher pKa and more positive charged positions had higher effects on both in vitro anti-malarial activity and anti-haemozoin activity compared to acridines with only one positive charged position. On the other hand, Vennerstrom et al. [34] also suspected that better correlation could occur if the accumulation of tested biquinolines in the food vacuoles was improved. However, there have not been any studies investigating whether the accumulation of biquinolines in the food vacuoles would positively reflect the in vitro anti-malarial activity or anti-haemozoin activity. Taken them together, there possibly was a clue of why not all β-haematin inhibitors can become potential anti-malarial candidates, as the accumulation of these inhibitors inside the food vacuoles resulted in varied efficacy on their anti-haemozoin effect which could fluctuate the anti-malarial activity. The investigation of their structure to improve their ability to accumulate in food vacuoles, thus, could be effective.

In contrast, there were still several studies reporting a satisfactory correlation between IC_50_ values of in vitro anti-malarial activity and IC_5o_ values of anti-haemozoin activity for some chloroquinolines (benzylated analogue, and deprotected analogue in which benzylate moiety was lyzed), quinolines (chloroquine, amodiaquine, pyronaridine, quinine, mefloquine, Ro 48-6910) and xanthones [22, 31, 35]. However, the good correlation for chloroquinoline deprotected analogues was just fortuitous, as the authors reported the R^2^ value remarkably decreased to 0.045 if an outlier point was omitted [22]. This means the in vitro anti-malarial activity and anti-haemozoin activity of chloroquines deprotected analogues were possibly not well correlated as the presented results. To confirm this, we suggest further studies performing their analysis with more data and removing outliers in data before analysis. Except the research of Andayi et al. [22], other reports showed that these tested compounds, namely some chloroquinolines, quinolines and xanthones had strong affinity with haem that could explain for the accumulation of their concentration in the food vacuoles [22, 31, 35]. Although there was limited evidence for this theory, it should be considered for further studies to investigate deeper by modifying the structure of compounds which strongly bind to haem and examining their accumulation as well as their anti-malarial activity. For instance, some quinolines, xanthones and several acridines were effective in both in vitro anti-malarial and anti-haemozoin activities in our review that had the strong binding to haem with the high association constant were reported to have good accumulation in the food vacuoles [24, 31, 35]. Burgess et al. [30] indicated that two dipyridyl chloroquinolines having better binding to haem also showed better accumulation in the food vacuoles as well. It is known that chloroquine significantly interacted with ferriprotoporphyrin IX by the interaction of its π–π complex with two haem µ-oxo dimers [37]. Nevertheless, the included studies exhibited that chloroquine analogues had a variety of the association constant values despite they shared the same scaffold [30–32]. This could refer to the role of moieties at the tertiary amino nitrogen in their chemical structure, but that has not been elucidated. Reinforcing this theory, the result from the study of Ignatushchenko et al. [35] on xanthone scaffold also disclosed hydroxyls at position 4-, and 5- were favored to the good correlation between anti-haemozoin activity and in vitro anti-malarial effects. These hydroxyls were able to sharply interact with haem by the establishment of soluble complexes. Polyhydroxyl xanthones (six moieties of hydroxyl) created even more interactions between haem iron and carbonyl oxygen, and between haem carboxylate side-groups and hydroxyls of xanthone. This is clearer, as xanthones that had hydroxyl radicals replaced by the acetyl radical lost its anti-haemozoin effects. However, there were some xanthones failing to inhibit the haemozoin formation but possessing strong in vitro anti-malarial effects as well, which raised their ability of another mechanism of anti-malarial activity.

Regarding the poor correlation between IC_50_ values of in vitro anti-malarial and anti-haemozoin activities for chloroquinolines in resistant strain K1, reported by Hawley et al. [23], this was explained by the decrease in the accumulation of chloroquinolines (amodiaquine, chloroquine, amodesethyl amodiaquine, *N*-tert-butyl amodiaquine, amopyroquine, 4′-dehydroxytebuquine) in food accumulation in the resistant strain K1. The vacuoles accumulation ratios in the resistant K1 were only one-third of that in the sensitive strain. So far, it was elucidated that resistant strains had a mutation in chloroquine transporter protein (PfCRT) which helped the parasites to eliminate the diprotonic forms of chloroquine accumulated in the organelle [37, 38]. Additionally, the modest to good correlations between anti-haemozoin and in vitro anti-malarial activity in resistant strain W2, for deprotected chloroquinolines and biquinolines, might imply that the resistance based on mutation of PfCRT only occurred for some specific compounds, not for all compounds of chloroquinoline class [22, 34].

Recent study continued to screen anti-malarial candidates from β-haematin inhibitors [33]. The results showed that quinolines, 4-benzamidopyridine, quinazoline, phenyl benzamides, nicotinamide, carbazole, and miscellaneous compounds had potential effects on both in vitro anti-malarial and anti-haemozoin activity. However, the correlation of these activities was not desirable for the scaffolds-evidence. No suggested explanation was revealed in this study. The drug accumulation in the parasite food vacuole is an important factor leading to its sufficient concentration to cause anti-haemozoin activity. This could be seen as there were many quinolines that had good anti-haemozoin activity but had inactive in vitro anti-malarial activity [23, 34]. The ability to accumulate in the food vacuole were also widely varied amongst compounds in quinoline scaffold [34]. Therefore, it was suggested that the differences in chemical properties of these different scaffolds in the study of Sandlin et al. [33] leading to the different manners in drug accumulation which resulted in the poor correlation. In addition, these classes would possibly have other mechanisms of action besides the anti-haemozoin activity which affected the correlation between anti-malarial and anti-haemozoin activities.

The limitation of the study is the small number of included studies of which there were only few recent findings. There are still gaps in the current knowledge of chloroquine resistance that caused obstructions in the discovery of new anti-malarial drugs. Due to limit data, the correlation between in vitro anti-malarial activity and anti-haemozoin activity has not been confirmed, especially when the analysis in this review removed compounds not possessing anti-malarial or anti-haemozoin effect. On top of that, there were few outliers in the analysis which had much higher IC_50_ values or BHIA_50_ values than other points. None of them was removed when calculating the correlation because using the same data as the original studies was better to make the suitable comparison. However, these outliers might affect the real correlation between in vitro anti-malarial and anti-haemozoin activities. Further studies should screen a bigger number of compounds in the same scaffolds and give attention to the accumulation of individual compounds when performing analysis.

## Conclusions

In vitro anti-malarial and anti-haemozoin activities had a good correlation for quinolines, chloroquinolines and xanthones. With the removals of some compounds which did not have either anti-malarial effect or anti-haemozoin effect, the good correlation between these activities reached satisfactory results in some cases. Some characteristics of compounds reinforcing the accumulation in the food vacuoles were highlighted in our review. Higher pKa value and the diprotonic form of chloroquine positively affected its concentration in the food vacuoles while acridines with higher pKa values appeared to have stronger anti-malarial and anti-haemozoin activities. In addition to the interaction between chloroquines and ferriprotoporphyrin IX via the π–π complex with two haem µ-oxo dimers, the moieties at the tertiary amino nitrogen in their chemical structure probably play an important role in the haem binding, although this has not been elucidated. Finally, the higher number hydroxyl moieties in xanthone scaffold might strengthen its efficacy of both anti-malarial activity and anti-haemozoin activity. These physiochemical properties helped their ability to accumulate inside the reaction site might strengthen their anti-malarial activity that should be remarkably considered when screening for new anti-malarial candidates in further studies.

## Supplementary information

**Additional file 1: Table S1.** PRISMA checklist.

**Additional file 2: Fig. S1.** Correlation between β-haematin inhibition activity (BIHA_50_, µM) and anti-malarial activity (IC_50_-, nM) for reversed chloroquinolines against sensitive strain D6. A compound which had strong anti-malarial activity (IC_50_ = 2 nM) was removed from the analysis due to its insoluble form in anti-haemozoin test.

**Additional file 3: Fig. S2.** Correlation between β-haematin inhibition activity (log(BIHA_50_)) and anti-malarial activity (log(IC_50_)-) for reversed chloroquinolines against sensitive strain D6. A compound which had strong anti-malarial activity (IC_50_ = 2 nM) was removed from the analysis due to its insoluble form in anti-haemozoin test.

**Additional file 4: Fig. S3.** Correlation between β-haematin inhibition activity (BIHA_50_, µM) and anti-malarial activity (IC_50_-, µM) for benzylate chloroquinolines against sensitive strain 3D7. Compound 8d was removed from this analysis due to its inactivation of haemozoin formation, despite it had good anti-malarial effect.

**Additional file 5: Fig. S4.** Correlation between β-haematin inhibition activity (log(BIHA_50_), µM) and anti-malarial activity (log(IC_50_)-) for benzylate chloroquinolines against sensitive strain 3D7. Compound 8d was removed from this analysis due to its inactivation of haemozoin formation, despite it had good anti-malarial effect.

**Additional file 6: Fig. S5.** Correlation between β-haematin inhibition activity (BIHA_50_, µM) and anti-malarial activity (IC_50_-, µM) for deprotected chloroquinolines against sensitive strain 3D7.

**Additional file 7: Fig. S6.** Correlation between β-haematin inhibition activity (log(BIHA_50_)) and anti-malarial activity (log(IC_50_)-) for deprotected chloroquinolines against sensitive strain 3D7.

**Additional file 8: Fig. S7.** Correlation between β-haematin inhibition activity (BIHA_50_, µM) and anti-malarial activity (IC_50_-, µM) for deprotected chloroquinolines against sensitive strain W2.

**Additional file 9: Fig. S8.** Correlation between β-haematin inhibition activity (log(BIHA_50_)) and anti-malarial activity (log(IC_50_)-) for deprotected chloroquinolines against sensitive strain W2.

**Additional file 10: Fig. S9.** Correlation between β-haematin inhibition activity (BIHA_50_, µM) and anti-malarial activity (IC_50_-, µM) for benzylate chloroquinolines against sensitive strain W2.

**Additional file 11: Fig. S10.** Correlation between β-haematin inhibition activity (log(BIHA_50_)) and anti-malarial activity (log(IC_50_)-) for benzylate chloroquinolines against sensitive strain W2.

**Additional file 12: Fig. S11.** Correlation between β-haematin inhibition activity (BIHA_50_, µM) and anti-malarial activity (IC_50_-, µM) for deprotected chloroquinolines against sensitive strain K1.

**Additional file 13: Fig. S12.** Correlation between β-haematin inhibition activity (log(BIHA_50_)) and anti-malarial activity (log(IC_50_)-) for deprotected chloroquinolines against sensitive strain K1.

**Additional file 14: Fig. S13.** Correlation between β-haematin inhibition activity (BIHA_50_, µM) and anti-malarial activity (IC_50_-, µM) for benzylate chloroquinolines against resistant strain K1. Compound 8d was removed from this analysis because of its non-detectable anti-haemozoin activity.

**Additional file 15: Fig. S14.** Correlation between β-haematin inhibition activity (log(BIHA_50_)) and anti-malarial activity (log(IC_50_)-) for benzylate chloroquinolines against resistant strain K1. Compound 8d was removed from this analysis because of its non-detectable anti-haemozoin activity.

**Additional file 16: Fig. S15.** Correlation between β-haematin inhibition activity (BIHA_50_, µM) and anti-malarial activity (IC_50_-, nM) for reversed chloroquinolines against resistant strain 7G8.

**Additional file 17: Fig. S16.** Correlation between β-haematin inhibition activity (log(BIHA_50_)) and anti-malarial activity (log(IC_50_)-) for reversed chloroquinolines against resistant strain 7G8.

**Additional file 18: Fig. S17.** Correlation between β-haematin inhibition activity (BIHA_50_, µM) and anti-malarial activity (IC_50_-, nM) for reversed chloroquinolines against resistant strain Dd2.

**Additional file 19: Fig. S18.** Correlation between β-haematin inhibition activity (log(BIHA_50_)) and anti-malarial activity (log(IC_50_)-) for reversed chloroquinolines against resistant strain Dd2.

**Additional file 20: Fig. S19.** Correlation between β-haematin inhibition activity (BIHA_50_, µM) and anti-malarial activity (normalized-IC_50_, nM) for quinolines against sensitive strain D10. There were three compounds (with hydro-, hydroxyl-, and acetyl- radicals at 7-position) were removed from this analysis, because they had very weak anti-malarial activity (IC_50_ ranging from 448 to 3017 nM) and non-detectable anti-haemozoin activity.

**Additional file 21: Fig. S20.** Correlation between β-haematin inhibition activity (BIHA_50_, µM) and anti-malarial activity (IC_50_-, nM) for biquinolines against sensitive strain W2.

**Additional file 22. Fig. S21.** Correlation between β-haematin inhibition activity (log(BIHA_50_)) and anti-malarial activity (log(IC_50_)-) for biquinolines against sensitive strain W2.

**Additional file 23. Fig. S22.** Correlation between β-haematin inhibition activity (BIHA_50_, µM) and anti-malarial activity (IC_50_-, nM) for biquinolines against sensitive strain W2 and D6.

**Additional file 24: Fig. S23.** Correlation between β-haematin inhibition activity (log(BIHA_50_)) and anti-malarial activity (log(IC_50_)-) for biquinolines against sensitive strain W2 and D6.

**Additional file 25: Fig. S24.** Correlation between β-haematin inhibition activity (BIHA_50_, µM) and anti-malarial activity (IC_50_-, nM) for biquinolines against sensitive strain D6.

**Additional file 26: Fig. S25.** Correlation between β-haematin inhibition activity (log(BIHA_50_)) and anti-malarial activity (log(IC_50_)-) for biquinolines against sensitive strain D6.

**Additional file 27: Fig. S26.** Correlation between β-haematin inhibition activity (BIHA_50_, µM) and anti-malarial activity (IC_50_-, µM) for xanthones against sensitive strain D6. 2-hydroxanthone, 1,3-dihydroxyxanthone, 2,3,4,5,6- pentaacetylxanthone were active against *P. falciparum* D6 strain (IC_50_ ranged from 0.075–75 µM) but did not possess the anti-haemozoin activity (IC_50_ > 1000 µM)

**Additional file 28: Fig. S27.** Correlation between β-haematin inhibition activity (log(BIHA_50_)) and anti-malarial activity (log(IC_50_)-) for xanthones against sensitive strain D6. 2-hydroxanthone, 1,3-dihydroxyxanthone, 2,3,4,5,6- pentaacetylxanthone were active against *P. falciparum* D6 strain (IC_50_- ranged from 0.075–75 µM) but did not possess the anti-haemozoin activity (IC_50_ > 1000 µM)

**Additional file 29: Fig. S28.** Correlation between β-haematin inhibition activity (BIHA_50_, µM) and anti-malarial activity (IC_50_-, µM) for acridines against sensitive strain 3D7.

**Additional file 30: Fig. S29.** Correlation between β-haematin inhibition activity (BIHA_50_, µM) and anti-malarial activity (IC_50_-, µM) for acridines against sensitive strain W2.

**Additional file 31: Fig. S30.** Correlation between β-haematin inhibition activity (BIHA_50_, µM) and anti-malarial activity (IC_50_-, µM) for acridines against resistant strain BRE1.

**Additional file 32: Fig. S31.** Correlation between β-haematin inhibition activity (BIHA_50_, µM) and anti-malarial activity (IC_50_-, µM) for acridines against resistant strain FCR3.

**Additional file 33: Fig. S32.** Correlation between β-haematin inhibition activity (BIHA_50_, µM) and anti-malarial activity (IC_50_-, µM) for quinoline, phenyl benzamides, 4-benzamidopyridine, quinazoline, carbazole, nicotinamide and miscellaneous compounds against sensitive strain D6.

**Additional file 34: Fig. S33.** Correlation between β-haematin inhibition activity (BIHA_50_, µM) and anti-malarial activity (IC_50_-, µM) for quinoline, phenyl benzamides, 4-benzamidopyridine, quinazoline, carbazole, nicotinamide and miscellaneous compounds against sensitive strain C235.

## Data Availability

The datasets used and/or analyzed during the current study are available from the corresponding author on reasonable request.

## Abbreviations

ACT: Artemisinin-based combination therapy
BHIA_50_: β-haematin inhibitory activity 50%
CQ: Chloroquine
IC_50_: The half maximal inhibitory concentration
GHL: WHO health library
PRISMA: Preferred Reporting Items for Systematic reviews and Meta-Analyses
SIGLE: System for Information on Grey Literature in Europe
VHL: Virtual Health Library
