# Supplementary material for: Correlation between anti-malarial and anti-haemozoin activities of anti-malarial compounds
Source: Malar J. 2020 Aug 21;19:298. doi: 10.1186/s12936-020-03370-x (PMC7441662; doi:10.1186/s12936-020-03370-x)
Supplement: Supplementary file 2 — Additional file 2: Fig. S1. Correlation between β-haematin inhibition activity (BIHA50, µM) and anti-malarial activity (IC50-, nM) for reversed chloroquinolines against sensitive strain D6. A compound which had strong anti-malarial activity (IC50 = 2 nM) was removed from the analysis due to its insoluble form in anti-haemozoin test. [file 12936_2020_3370_MOESM2_ESM.pptx]

## Slide 1
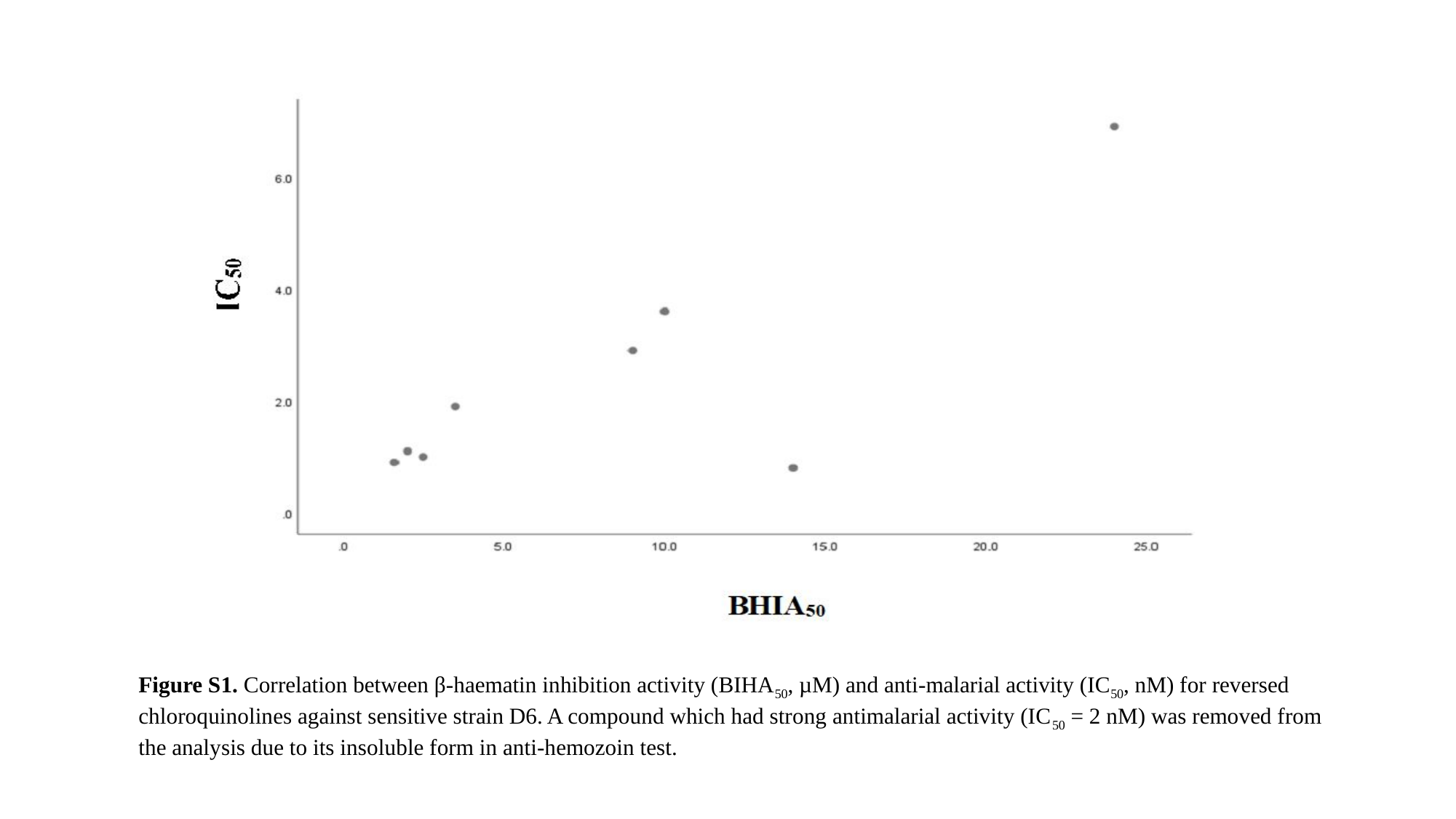

#
Figure S1. Correlation between β-haematin inhibition activity (BIHA50, µM) and anti-malarial activity (IC50­, nM) for reversed chloroquinolines against sensitive strain D6. A compound which had strong antimalarial activity (IC50 = 2 nM) was removed from the analysis due to its insoluble form in anti-hemozoin test.
