# Supplementary material for: Correlation between anti-malarial and anti-haemozoin activities of anti-malarial compounds
Source: Malar J. 2020 Aug 21;19:298. doi: 10.1186/s12936-020-03370-x (PMC7441662; doi:10.1186/s12936-020-03370-x)
Supplement: Supplementary file 3 — Additional file 3: Fig. S2. Correlation between β-haematin inhibition activity (log(BIHA50)) and anti-malarial activity (log(IC50)-) for reversed chloroquinolines against sensitive strain D6. A compound which had strong anti-malarial activity (IC50 = 2 nM) was removed from the analysis due to its insoluble form in anti-haemozoin test. [file 12936_2020_3370_MOESM3_ESM.pptx]

## Slide 1
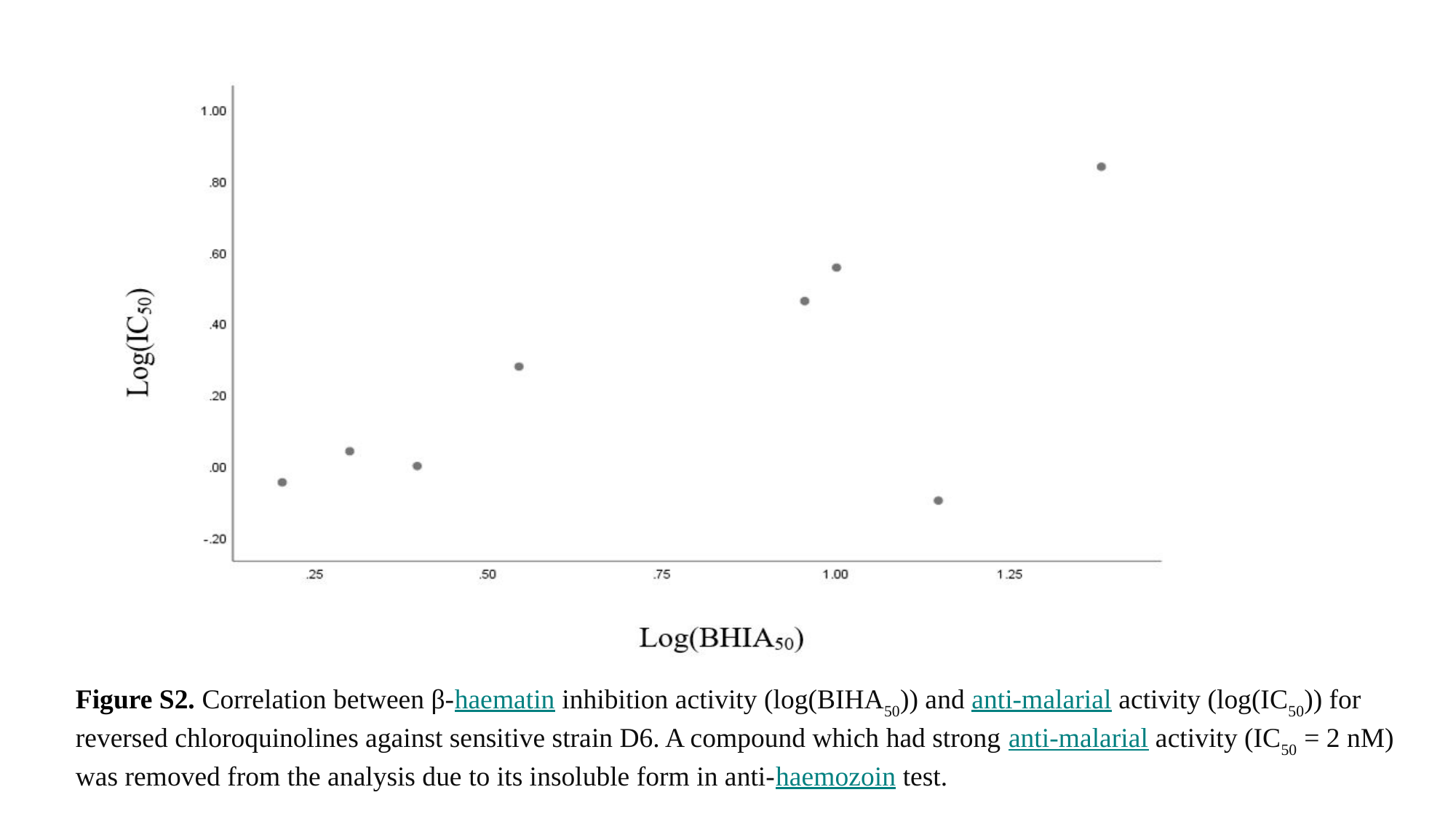

Figure S2. Correlation between β-haematin inhibition activity (log(BIHA50)) and anti-malarial activity (log(IC50)­) for reversed chloroquinolines against sensitive strain D6. A compound which had strong anti-malarial activity (IC50 = 2 nM) was removed from the analysis due to its insoluble form in anti-haemozoin test.
