# Supplementary material for: Correlation between anti-malarial and anti-haemozoin activities of anti-malarial compounds
Source: Malar J. 2020 Aug 21;19:298. doi: 10.1186/s12936-020-03370-x (PMC7441662; doi:10.1186/s12936-020-03370-x)
Supplement: Supplementary file 4 — Additional file 4: Fig. S3. Correlation between β-haematin inhibition activity (BIHA50, µM) and anti-malarial activity (IC50-, µM) for benzylate chloroquinolines against sensitive strain 3D7. Compound 8d was removed from this analysis due to its inactivation of haemozoin formation, despite it had good anti-malarial effect. [file 12936_2020_3370_MOESM4_ESM.pptx]

## Slide 1
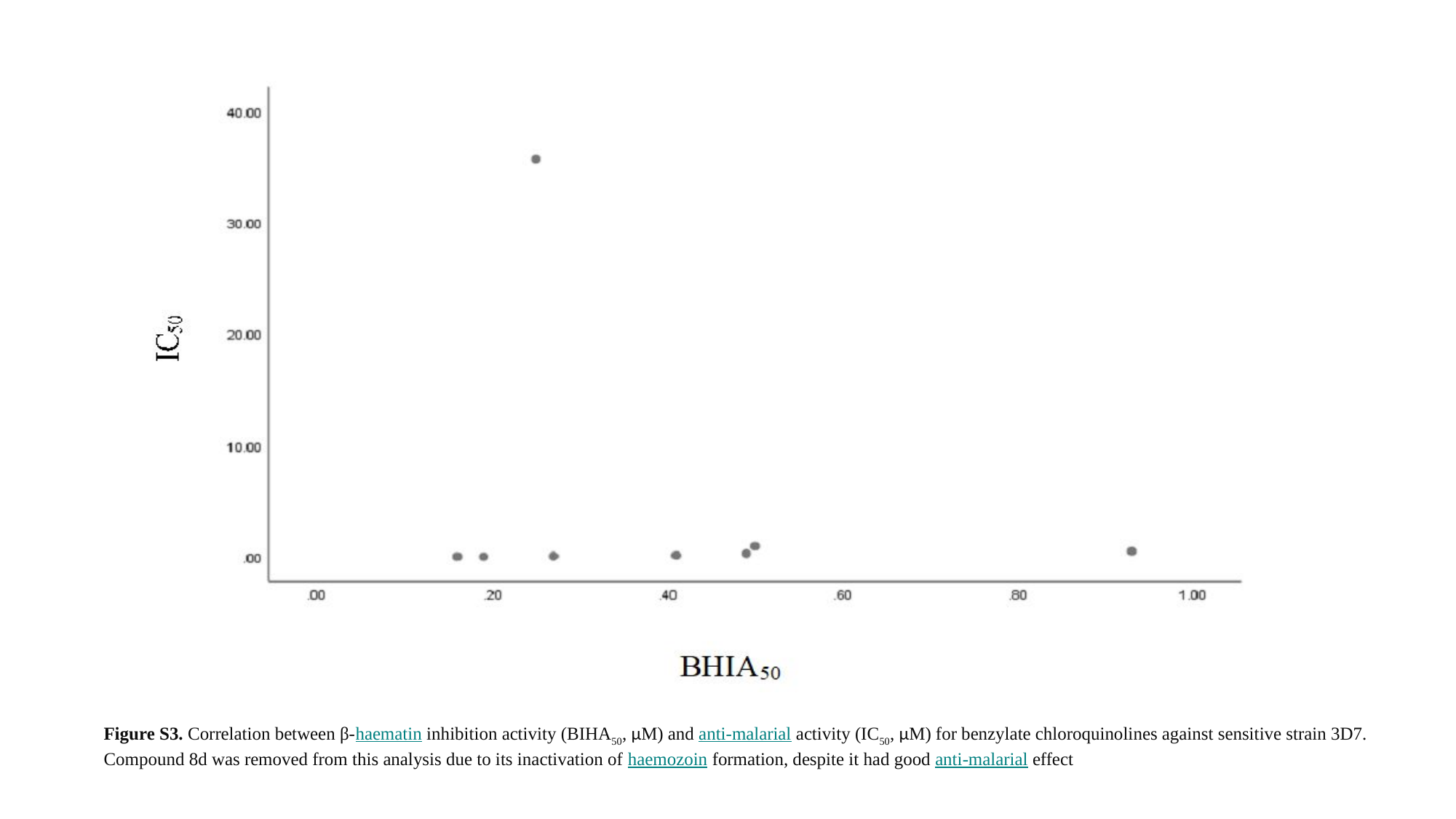

#
Figure S3. Correlation between β-haematin inhibition activity (BIHA50, µM) and anti-malarial activity (IC50­, µM) for benzylate chloroquinolines against sensitive strain 3D7. Compound 8d was removed from this analysis due to its inactivation of haemozoin formation, despite it had good anti-malarial effect
