# Supplementary material for: Correlation between anti-malarial and anti-haemozoin activities of anti-malarial compounds
Source: Malar J. 2020 Aug 21;19:298. doi: 10.1186/s12936-020-03370-x (PMC7441662; doi:10.1186/s12936-020-03370-x)
Supplement: Supplementary file 8 — Additional file 8: Fig. S7. Correlation between β-haematin inhibition activity (BIHA50, µM) and anti-malarial activity (IC50-, µM) for deprotected chloroquinolines against sensitive strain W2. [file 12936_2020_3370_MOESM8_ESM.pptx]

## Slide 1
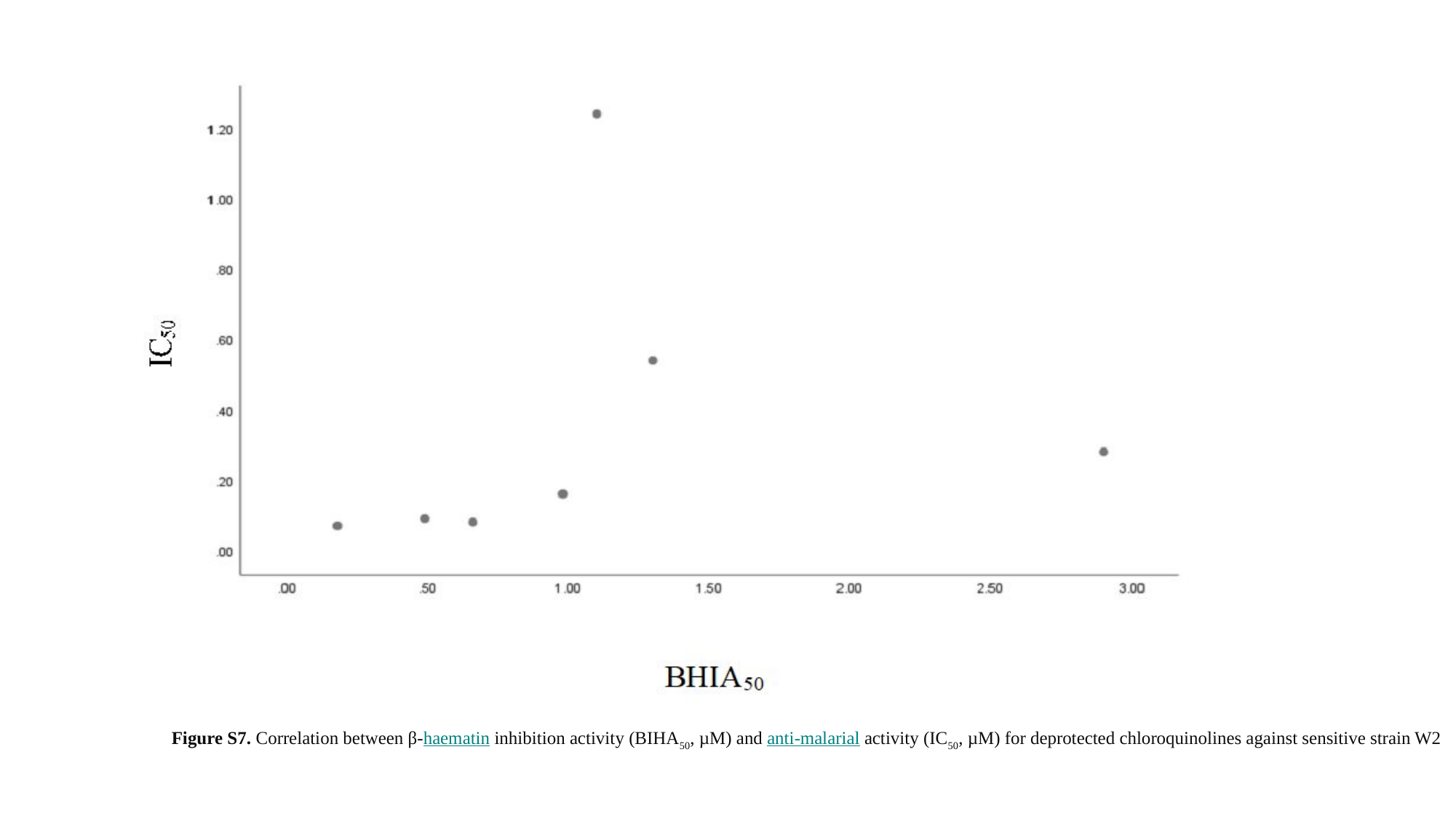

#
Figure S7. Correlation between β-haematin inhibition activity (BIHA50, µM) and anti-malarial activity (IC50­, µM) for deprotected chloroquinolines against sensitive strain W2
