# Supplementary material for: Correlation between anti-malarial and anti-haemozoin activities of anti-malarial compounds
Source: Malar J. 2020 Aug 21;19:298. doi: 10.1186/s12936-020-03370-x (PMC7441662; doi:10.1186/s12936-020-03370-x)
Supplement: Supplementary file 17 — Additional file 17: Fig. S16. Correlation between β-haematin inhibition activity (log(BIHA50)) and anti-malarial activity (log(IC50)-) for reversed chloroquinolines against resistant strain 7G8. [file 12936_2020_3370_MOESM17_ESM.pptx]

## Slide 1
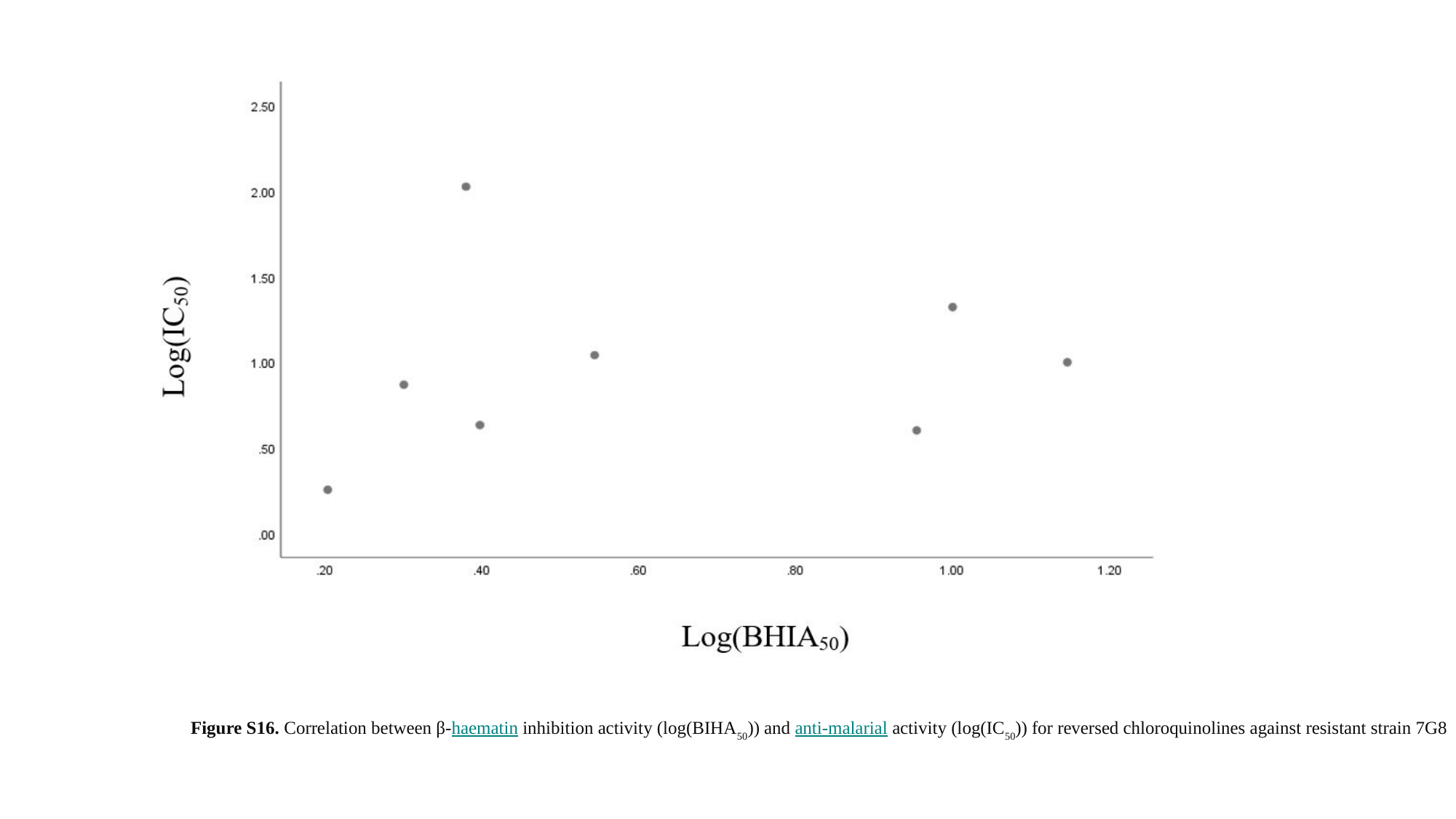

Figure S16. Correlation between β-haematin inhibition activity (log(BIHA50)) and anti-malarial activity (log(IC50­)) for reversed chloroquinolines against resistant strain 7G8
