# Supplementary material for: Correlation between anti-malarial and anti-haemozoin activities of anti-malarial compounds
Source: Malar J. 2020 Aug 21;19:298. doi: 10.1186/s12936-020-03370-x (PMC7441662; doi:10.1186/s12936-020-03370-x)
Supplement: Supplementary file 18 — Additional file 18: Fig. S17. Correlation between β-haematin inhibition activity (BIHA50, µM) and anti-malarial activity (IC50-, nM) for reversed chloroquinolines against resistant strain Dd2. [file 12936_2020_3370_MOESM18_ESM.pptx]

## Slide 1
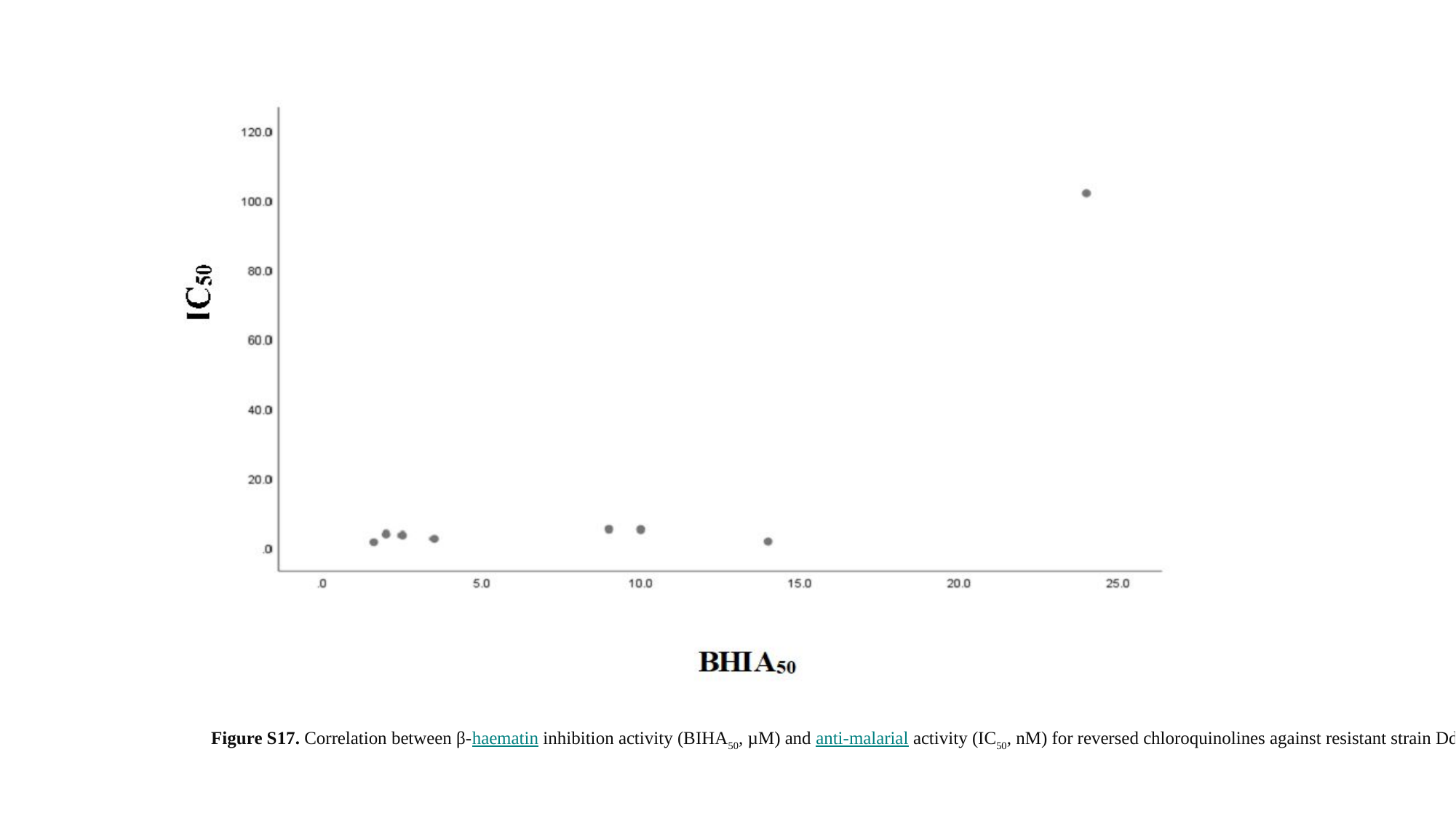

Figure S17. Correlation between β-haematin inhibition activity (BIHA50, µM) and anti-malarial activity (IC50­, nM) for reversed chloroquinolines against resistant strain Dd2
