# Supplementary material for: Correlation between anti-malarial and anti-haemozoin activities of anti-malarial compounds
Source: Malar J. 2020 Aug 21;19:298. doi: 10.1186/s12936-020-03370-x (PMC7441662; doi:10.1186/s12936-020-03370-x)
Supplement: Supplementary file 20 — Additional file 20: Fig. S19. Correlation between β-haematin inhibition activity (BIHA50, µM) and anti-malarial activity (normalized-IC50, nM) for quinolines against sensitive strain D10. There were three compounds (with hydro-, hydroxyl-, and acetyl- radicals at 7-position) were removed from this analysis, because they had very weak anti-malarial activity (IC50 ranging from 448 to 3017 nM) and non-detectable anti-haemozoin activity. [file 12936_2020_3370_MOESM20_ESM.pptx]

## Slide 1
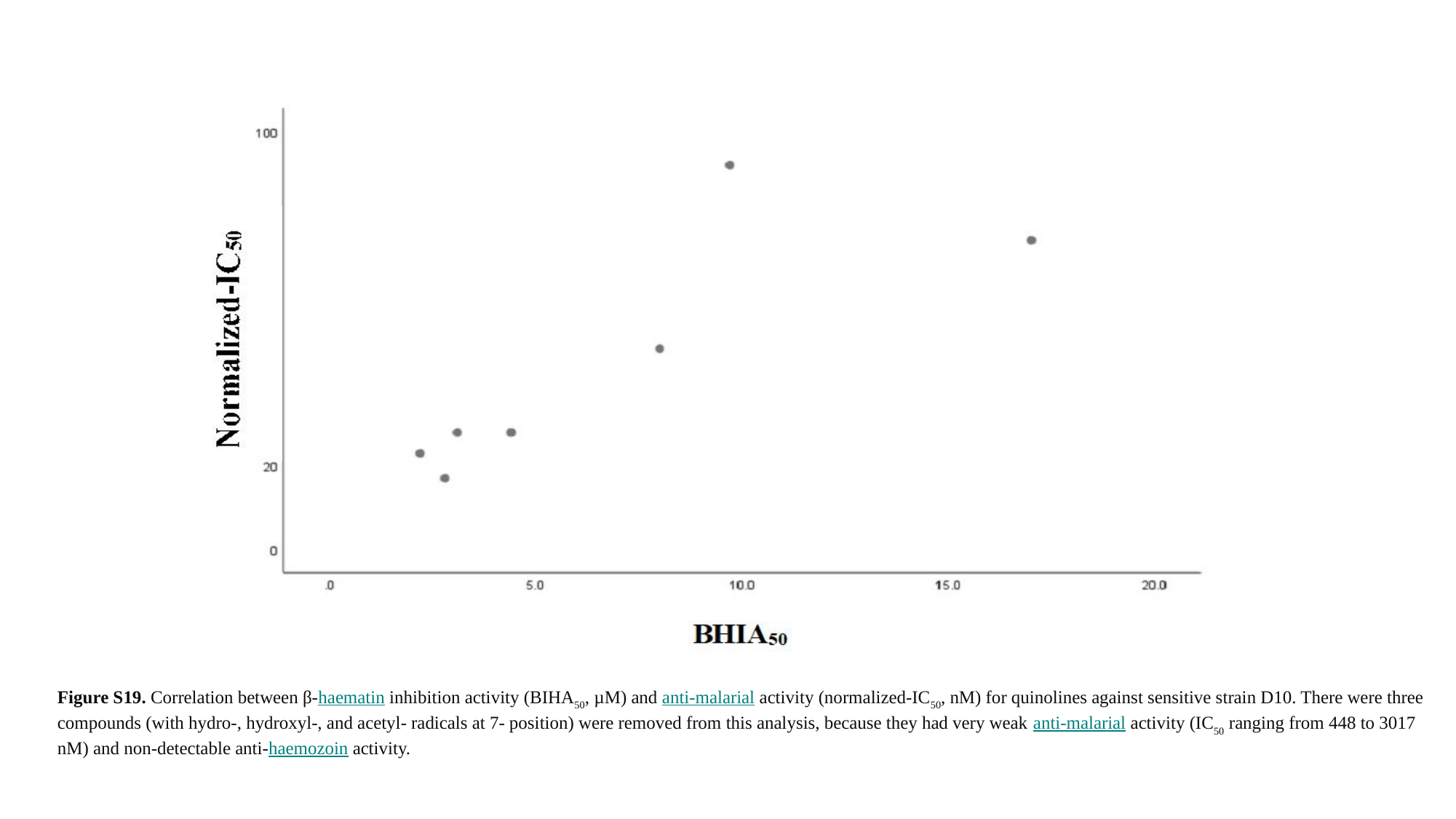

Figure S19. Correlation between β-haematin inhibition activity (BIHA50, µM) and anti-malarial activity (normalized-IC50­, nM) for quinolines against sensitive strain D10. There were three compounds (with hydro-, hydroxyl-, and acetyl- radicals at 7- position) were removed from this analysis, because they had very weak anti-malarial activity (IC50 ranging from 448 to 3017 nM) and non-detectable anti-haemozoin activity.
