# Supplementary material for: Correlation between anti-malarial and anti-haemozoin activities of anti-malarial compounds
Source: Malar J. 2020 Aug 21;19:298. doi: 10.1186/s12936-020-03370-x (PMC7441662; doi:10.1186/s12936-020-03370-x)
Supplement: Supplementary file 27 — Additional file 27: Fig. S26. Correlation between β-haematin inhibition activity (BIHA50, µM) and anti-malarial activity (IC50-, µM) for xanthones against sensitive strain D6. 2-hydroxanthone, 1,3-dihydroxyxanthone, 2,3,4,5,6- pentaacetylxanthone were active against P. falciparum D6 strain (IC50 ranged from 0.075–75 µM) but did not possess the anti-haemozoin activity (IC50 > 1000 µM) [file 12936_2020_3370_MOESM27_ESM.pptx]

## Slide 1
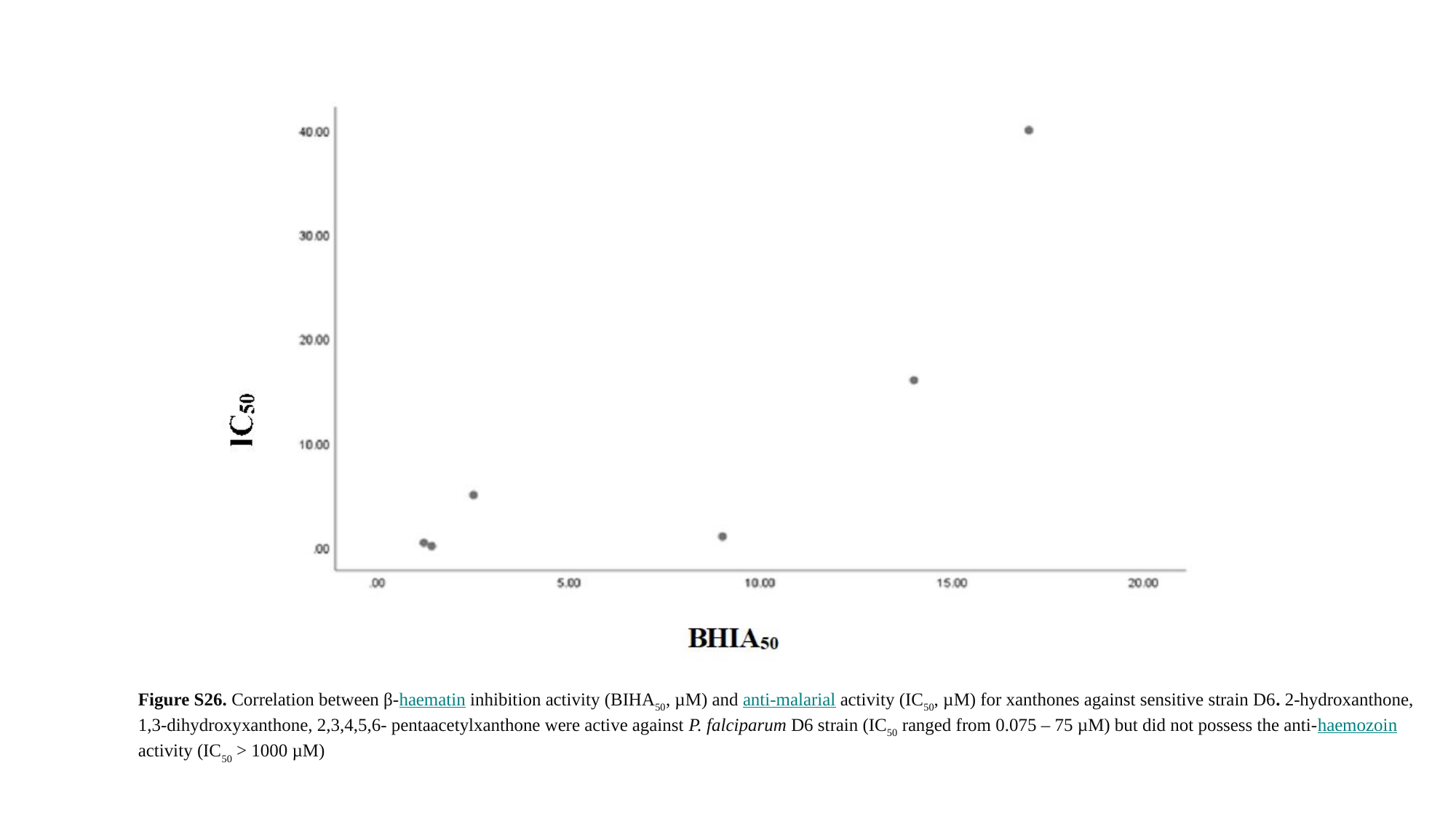

Figure S26. Correlation between β-haematin inhibition activity (BIHA50, µM) and anti-malarial activity (IC50­, µM) for xanthones against sensitive strain D6. 2-hydroxanthone, 1,3-dihydroxyxanthone, 2,3,4,5,6- pentaacetylxanthone were active against P. falciparum D6 strain (IC50 ranged from 0.075 – 75 µM) but did not possess the anti-haemozoin activity (IC50 > 1000 µM)
